# Noticeable effect of lower baseline amplitude on the predictive accuracy of intraoperative amplitude changes for postoperative vocal cord palsy: a prospective cohort study

**DOI:** 10.1097/JS9.0000000000001203

**Published:** 2024-02-21

**Authors:** Jiedong Kou, Yishen Zhao, Yujia Han, Fang Li, Rui Du, Gianlorenzo Dionigi, Francesco Frattini, Jingting Li, Nan Liang, Hui Sun

**Affiliations:** a Division of Thyroid Surgery, The China-Japan Union Hospital of Jilin University, Jilin Provincial Key Laboratory of Surgical Translational Medicine, Jilin Provincial Precision Medicine Laboratory of Molecular Biology and Translational Medicine on Differentiated Thyroid Carcinoma, Changchun City, Jilin Province, People’s Republic of China; b Department of Medical Biotechnology and Translational Medicine, Division of General and Endocrine Surgery, Istituto Auxologico Italiano IRCCS, University of Milan, Milan, Italy

**Keywords:** amplitude, intraoperative nerve monitoring, morbidity, recurrent laryngeal nerve, thyroid surgery, vocal cord palsy

## Abstract

**Background:**

To explore the effect of lower baseline amplitude on its predictive accuracy of postoperative vocal cord paralysis (VCP) in monitored thyroid surgery.

**Materials and methods:**

Clinical and electrophysiological data were collected during thyroid surgeries performed between November and December 2021 at China-Japan Union Hospital. Univariate/multivariate regression analysis were applied to these data to examine a possible correlation. A receiver operating characteristic curve was used to evaluate predictive efficacy.

**Results:**

A total of 631 nerves-at-risk (NAR) were identified in 460 patients who were divided into two groups according to postoperative development of VCP. The VCP group included a higher percentage of NAR with V1<1000 (68.2 vs. 40.7%, respectively; *P*=0.014) and NAR with R1<1400 (77.3 vs. 47.0%, respectively; *P*=0.005) compared with the non-VCP group. Multivariate regression analysis further identified V1<1000 [odds ratio (OR)=2.688, *P*=0.038], R1<1400 (OR=3.484, *P*=0.018) as independent risk factors for postoperative temporary VCP. The receiver operating characteristic curve showed the AUC value of V signal decline for predicting VCP was 0.87. The diagnostic efficiency of R signal decline reached as high as 0.973. A multivariate logistic regression analysis identified independent risk factors for V1<1000 and these included: higher BMI (OR=1.072, *P*=0.013), hypertension (OR=1.816, *P*=0.015), smoking (OR=1.814, *P*=0.031), and male sex (OR=2.016, *P*=0.027).

**Conclusion:**

In our cohort, lower baseline amplitude was an independent risk factor for developing transient postoperative VCP. It also affected the predictive efficacy of intraoperative amplitude changes on VCP. Higher BMI, hypertension, smoking, and male sex may also be closely associated with lower initial amplitude. Thus, maintaining a higher initial amplitude is critical for patient safety during thyroid surgery.

## Introduction

Highlights

- This study first explored a possible correlation between the initial amplitude of nerve electromiogram signal and postoperative temporary vocal cord paralysis (VCP), and we found that lower baseline amplitude was an independent risk factor for developing transient postoperative VCP. It also affected the predictive efficacy of intraoperative amplitude changes on VCP.
- The receiver operating characteristic curve of intraoperative amplitude decline in predicting the occurrence of postoperative temporary VCP showed that the value of R signal decline was more predictive of postoperative transient VCP than the decline in intraoperative V signal. The importance of intraoperative real-time monitoring and comparison of recurrent laryngeal nerve amplitude changes was confirmed.
- We found that male sex, smoking, combined hypertension, and higher BMI are factors, which influence a lower initial amplitude. This suggest that in clinical practise, the surgeon should be vigilant in patients with these characteristics and try to communicate fully with the anesthetist to achieve high initial amplitudes.

One of the most common complications of thyroid surgery involves injury to the recurrent laryngeal nerve (RLN)^1^. While, the reported incidence of RLN injury varies greatly depending on the examination methods used, a high rate of RLN injury has been observed at many medical institutions^2^. Therefore, intraoperative nerve monitoring (IONM) of the RLN has become an important component of thyroid surgeries^3–6^. Maintaining an adequate amplitude and latency profile for the RLN is critical for patient safety^7^. For example, when amplitude decreases and latency increases, the RLN is more susceptible to stress. If the surgical strategy remains unchanged, the nerve may be damaged^8,9^. Consequently, accurate monitoring of amplitude and latency is needed to guide RLN dissection, and also to ensure a safe and stable surgical environment.

An electromiogram (EMG) comprehensively reflects nerve status. Consequently, monitoring of the RLN based on EMG amplitude is a widely used method^10–13^. Therefore, it is important to start with appropriate reference values for the EMG signal amplitude of the RLN at the beginning of the surgical procedure. In addition, external influences can affect the amplitude achieved, such as the position of the inserted tube, tracheal shift, the type and dose of muscle relaxant administered, etc.^14,15^. Various physiological indicators of the patient are also considered. Therefore, the experience level and proficiency exhibited by an anesthesiologist can be relevant.

In the present study, we analyzed the amplitudes between different peak-to-peak bands of EMG signals to investigate whether low baseline amplitude is an index of increased susceptibility to RLN injury. Specific factors influencing low baseline amplitude were also examined.

## Materials and methods

### Study design

Single-centered prospective cohort study. The work has been reported in line with the strengthening the reporting of cohort studies in surgery (STROCSS) criteria^16^ (Supplemental Digital Content 1, http://links.lww.com/JS9/B1000).

### Time frame and setting

November through December 2021 at China-Japan Union Hospital of Jilin University.

### Ethical approval

The study protocol was approved by the Ethics Committee of China-Japan Union Hospital, Jilin University (No.2023020719). All of the patients signed an informed consent form prior to surgery. Written informed consent was obtained from the patient for publication of this case report and accompanying images. A copy of the written consent is available for review by the Editor-in-Chief of this journal on request.

### Cohort examined

Clinical data were collected from patients who underwent elective thyroid surgery with IONM. The need for thyroid surgery and the extent of thyroidectomy performed were determined according to guidelines of the American Thyroid Association^17^. Surgeries were performed by experienced surgeons who perform more than 800 operations per year.

### Inclusion criteria

Inclusion criteria for this study were: primary thyroid surgery performed, use of a standardized IONM technique during surgery, and a laryngeal examination performed before and after surgery. A postoperative laryngoscopy was conducted on the second day after surgery, and the findings were used as diagnostic criteria for vocal cord paralysis (VCP)^18^. All of the patients were ≥18 years old.

### Exclusion criteria

Patients were excluded from this study if their data were incomplete, if surgery was conducted on the parathyroid glands, if only cervical lymph node dissection was performed, if robotic and endoscopic thyroid surgery was performed, or if their preoperative laryngoscopy results were abnormal.

### Recording of EMG, anesthesia, and procedure performed

General anesthesia was performed according to the standard protocol of IONM. Briefly, anesthesia was induced with administration of propofol and remifentanil. Cisatracurium was subsequently administered to facilitate tracheal intubation. An endotracheal tube (ET) with surface electrodes (Medtronic) was placed in contact with the true vocal cords for intubation (size 6.0 or 7.0 tubing used). IONM was performed by using the NIM-Neuro 3.0 system (Medtronic). After the patient’s neck was stretched and its position adjusted, adequate contact between the electrodes and the vocal cords was confirmed by re-examining the larynx and checking EMG signals from the Nerve Monitor System (Supplementary Tables 1–3, Supplemental Digital Content 2, http://links.lww.com/JS9/C2). During surgery, connections were made according to standard surgical procedures for nerve monitoring^19–21^. Intermittent stimulations were applied to assess the integrity of nerve function, and an EMG was recorded from the surface electrode of the ET. The initial event threshold was 100 μV. After measuring the initial nerve amplitude, the event threshold for surgery was set at 50% of the initial amplitude.

Standard neurological monitoring steps were strictly followed throughout the procedure, including the intraoperative core method^19–21^. V1 signals were detected from the vagus nerve (VN) through the bulb-head stimulation probe with 3.0 mA in the surgical field before the start of surgery. R1 signals were obtained with 1.0 mA during initial detection of the RLN. R2 and V2 signals were obtained by detecting RLN and VN, respectively, with the same method applied upon completion of the procedures in the surgical area. The above data were recorded in real-time during surgery.

During thyroid surgery, surgical scalpel was used to cut the skin, and then electrotome (Force FX-8C, COVIDIEN MEDTRONIC) was used to open the skin flap. After opening the space of strap muscle, we perform the operation using an ultrasonic scalpel (HARMONIC FOCUS+ Shears, Johnson & Johnson). During the entire process of nerve detection, no electrotome was activated. The blood vessels were treated by using the ultrasonic scalpel to coagulate and/or ligate them. During the use of the ultrasonic scalpel, the activating side was always kept away from the nerves and a safe distance was maintained to avoid thermal injury. All patients underwent surgery following these methods and adhered to standardized steps in accordance with international guidelines for neural monitoring.

### Outcome measures

The following patient characteristics were recorded: sex, age, BMI, underlying diseases, and smoking history. Surgical characteristics were also recorded: type of surgery, type of thyroidectomy, intraoperative RLN findings, and RLN injuries. In addition, perioperative data, including duration of anesthesia and surgery, and postoperative laryngeal data were collected.

### Amplitude recording

The amplitude of an EMG signal is generally interpreted as an indicator of the activity level of the underlying muscle. According to international guidelines^19^, the surgeon and anesthesiologist collaborate to ensure that the baseline amplitude is as high as possible. The amplitude between different peak-to-peak bands of EMG signals was recorded for the surgeries performed. We defined a low baseline amplitude as V1<1000 μV (V1<1000) or R1<1400 μV (R1<1400). These values were selected because they represent the cut-off values obtained by statistical methods (see below).

### Postoperation follow-up

The results of laryngoscopy on the second day after operation showed that 22 patients had postoperative unilateral vocal cord paralysis. All these patients were followed up at 1 month, 3 months, and 6 months after surgery, including color Doppler ultrasound examination (including laryngeal vocal cords) and laryngoscopy. In this study, 22 patients were still in contact after discharge, and some patients came to the outpatient clinic for examination except the specified follow-up date to report the voice recovery to the doctor.

### Statistical methods

Statistical analyses were conducted using IBM SPSS 23.0,and charts were generated using GraphPad Prism 7.0. Arithmetic mean, SD, and median values were used to describe continuous variables. Weighted frequency and percentage (%) were used to describe categorical variables. The latter were analyzed using the Pearson *χ* ^2^ test, Fisher’s test, and Kruskal–Wallis *H* test. To analyze measurement data, the *t*-test, Mann–Whitney *U*-test, and Kruskal–Wallis *H* test were applied. In the univariate analysis performed, factors with an α-level of 0.05 were included in a multifactorial regression model. Differences with *P*-values<0.05 were considered statistically significant.

## Results

### Study population and VCP

Among the 460 patients enrolled in this study (335 females, 125 males) with a mean age of 44 years, there were 631 nerves-at-risk (NAR) identified. Of the cases involved in this study, 171 patients underwent total thyroidectomy and 289 underwent complete lobectomy, of which 139 were left side and 150 were right side. Unilateral VCP affected 22 (3.5%) of these NAR. There were no cases of bilateral VCP. The vocal cords function of 20 patients returned to normal within 1 to 3 months after operation, with an average recovery time of 46 days (the earliest 3 days, the latest 90 days). Another two cases who underwent nerve transection and nerve anastomosis due to tumor invasion did not recover vocal cord motion.

### Baseline amplitude risk stratification

To explore a possible correlation between the initial amplitude of nerve EMG signal and postoperative temporary VCP, the 631 NAR identified were divided into a VCP group (*n*=22) and a non-VCP group (*n*=609) based on postoperative laryngoscopies. The tangent point value with an appropriate initial amplitude was determined based on grouping of V1 according to the cut-off value obtained by ROC curve, median, mean, interquartile interval method, and values obtained by statistical screening. There were a greater number of nerves with V1<1000 in the VCP group than in the non-VCP group (68.2 vs. 40.7%, respectively; *P*=0.014) (Supplementary Table 4, Supplemental Digital Content 2, http://links.lww.com/JS9/C2). For all of the other grouping criteria, a trend of lower neural V1 signal was observed in the VCP group. Similarly, after grouping according to the cut-off value obtained by ROC curve, interquartile, and statistical screening value, there was a greater percentage of nerves with R1<1400 in the VCP group compared with the non-VCP group (77.3 vs. 47%, respectively; *P*=0.005). Scatter plots (Supplementary Figure 1, Supplemental Digital Content 2, http://links.lww.com/JS9/C2) further confirmed that the initial amplitude of the V1 and R1 signals tended to be lower in the VCP group. Therefore, based on statistical screening, V1=1000 and R1=1400 were selected as grouping criteria for subsequent analyses.

### Independent predictive value of baseline amplitude for VCP

**Figure 1:**
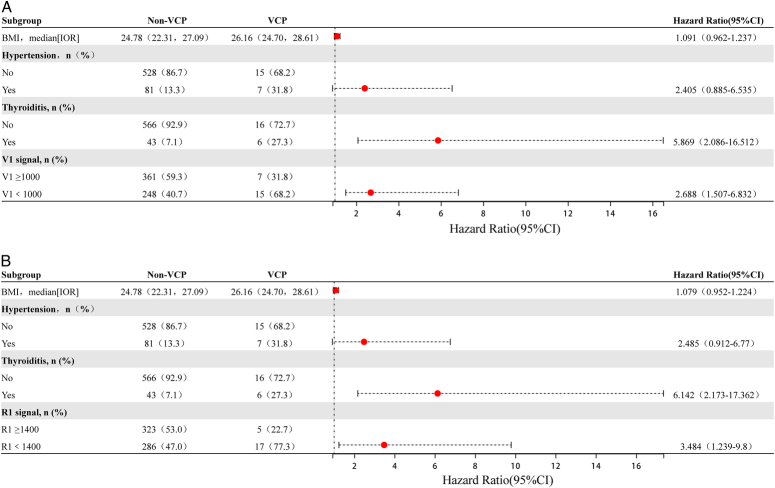
Multivariate Logistic regression forest plots describing the independent risk factors of VCP. To investigate the independent risk factors of VCP, the forest plots of multifactor regression analysis is as shown in Figure 1A, B. Since the RLN originates from the VN, V1 and R1 reflect the same electrophysiological conduction pathway of the nerve, given this collinearity, V1=1000 and R1=1400 were each included in a multivariate analysis model with other factor.

To investigate the predictive value of the initial amplitude for postoperative VCP, we first analyzed possible risk factors. As shown in Table 1, the VCP group had a higher BMI (26.16 kg/m^2^ vs. 24.78 kg/m^2^, respectively; *P*=0.021) and a higher proportion of overweight and obese patients (72.7 vs. 45.6%, respectively; *P*=0.015) than the non-VCP group. The VCP group also exhibited a higher incidence of thyroiditis (27.3 vs. 7.1%, respectively; *P*=0.005) and hypertension (31.8 vs. 13.3%, respectively; *P*=0.024). In a multivariate logistic regression analysis model, V1<1000 (OR=2.688, 95% CI=1.507–6.832, *P*=0.038), R1<1400 (OR=3.484, 95% CI=1.239–9.8, *P*=0.018), and combined thyroiditis (OR=5.869, 95% CI=2.086–16.512, *P*=0.001) were identified as independent risk factors (Table 2, Fig. 1A, B). Specifically, the risk of postoperative temporary VCP in patients with V1<1000 was 2.7 times that of patients with V1 ≥1000, while the risk of postoperative temporary VCP in patients with R1<1400 was 3.5 times that of patients with R1 ≥1400. Meanwhile, the risk of postoperative temporary VCP in patients with thyroiditis was approximately six times that of patients without thyroiditis. The above results further confirm that a lower initial amplitude is an independent risk factor for postoperative temporary VCP.

**Table 1:** Analysis of influencing factors of vocal cord palsy.

	Non-VCP (*n*=609)	VCP (*n*=22)		*P*		Non-VCP (*n*=609)	VCP (*n*=22)			*P*
Age (years),median [IOR]	45 (36.5–52)	45.5 (40.75–54.5)	Z=−0.573	0.566	Largest tumor size (cm),median [IOR]	0.99 (0.69–1.82)	0.82 (0.66–1.43)	Z=−1.062		0.288
Age (years), *n* (%)					V1 signal, *n* (%)					
≤55	515 (84.6)	17 (77.3)	Fisher	0.368	<1000	248 (40.7)	15 (68.2)	*χ* ^2^=6.586		0.014
>55	94 (15.4)	5 (22.7)			≥1000	361 (59.3)	7 (31.8)			
Sex, *n* (%)					R1 signal, *n* (%)					
Female	455 (74.7)	19 (86.4)	*χ* ^2^=1.542	0.315	<1400	286 (47.0)	17 (77.3)	*χ* ^2^=7.815		0.005
Male	154 (25.3)	3 (13.6)			≥1400	323 (53.0)	5 (22.7)			
Height (cm),median [IOR]	163 (160–170)	160.5 (158.3–165.3)	Z=−1.48	0.139	Operation time (min), median [IOR]	100 (79.5–130)	114.5 (90.5–136.3)	Z=−1.557		0.12
Weight (kg),median [IOR]	65 (58–75)	69 (65.3–75.5)	Z=−1.175	0.24	Postoperative pathology, *n* (%)					
BMI, median [IOR]	24.78 (22.31–27.09)	26.16 (24.70–28.61)	Z=−2.3	0.021	Benign	60 (9.9)	0 (0)	Fisher		0.254
BMI, *n* (%)					Malignant	549 (90.1)	22 (100)			
<18.5	10 (1.6)	0 (0)	Z=−2.435	0.015	T stage, *n* (%)					
18.5–24.99	321 (52.7)	6 (27.3)			T1+T2	527 (96.0)	21 (95.5)	Fisher		0.602
25–29.99	225 (36.9)	13 (59.1)			T3+T4	22 (4.0)	1 (4.5)			
≥30	53 (8.7)	3 (13.6)			N stage, *n* (%)					
Thyroiditis, *n* (%)					N0	307 (55.9)	11 (50.0)	*χ* ^2^=0.3		0.584
No	566 (92.9)	16 (72.7)	Fisher	0.005	N1	242 (44.1)	11 (50.0)			
Yes	43 (7.1)	6 (27.3)			M stage, *n* (%)					
Hyperthyreosis, *n* (%)					M0	547 (99.6)	22 (100.0)	Fisher		1.000
No	589 (96.7)	22 (100)	Fisher	1.000	M1	2 (0.4)	0 (0.0)			
Yes	20 (3.3)	0 (0)			AJCC stage, *n* (%)					
Smoking history, *n* (%)					I	530 (96.5)	20 (90.9)	Fisher		0.191
No	531 (87.2)	19 (86.4)	Fisher	0.754	II	19 (3.5)	2 (9.1)			
Yes	78 (12.8)	3 (13.6)			Multifocality, *n* (%)					
Hypertension, *n* (%)					No	339 (61.7)	10 (45.5)	*χ* ^2^=2.363		0.124
No	528 (86.7)	15 (68.2)	Fisher	0.024	Yes	210 (38.3)	12 (54.5)			
Yes	81 (13.3)	7 (31.8)			Extrathyroidal extension, *n* (%)					
Diabetes, *n* (%)					No	419 (76.3)	16 (72.7)	*χ* ^2^=0.151		0.698
No	578 (94.9)	20 (90.9)	Fisher	0.321	Yes	130 (23.7)	6 (27.3)			
Yes	31 (5.1)	2 (9.1)								

VCP, vocal cord palsy.

**Table 2:** Explore independent risk factors of vocal cord palsy based on multivariate logistic regression analysis.

		B	Standard error	Wald	OR (95% CI)	*P*
Model 1						
Constant term		6.561	1.707	14.775	0.001	0.000
BMI		0.087	0.064	1.834	1.091 (0.962–1.237)	0.176
Hypertension	Yes vs No	0.878	0.510	2.960	2.405 (0.885–6.535)	0.085
Thyroiditis	Yes vs No	1.770	0.528	11.246	5.869 (2.086–16.512)	0.001
V1 signal	<1000 vs ≥1000	0.989	0.476	4.313	2.688 (1.507–6.832)	0.038
Model 2						
Constant term		6.547	1.697	14.880	0.001	0.000
BMI		0.076	0.064	1.417	1.079 (0.952–1.224)	0.234
Hypertension	Yes vs No	0.910	0.511	3.168	2.485 (0.912–6.77)	0.075
Thyroiditis	Yes vs No	1.815	0.530	11.720	6.142 (2.173–17.362)	0.001
R1 signal	<1400 vs ≥1400	1.248	0.528	5.598	3.484 (1.239–9.8)	0.018

OR, odds ratio.

### Lower baseline amplitude affects surgical strategy

**Figure 2:**
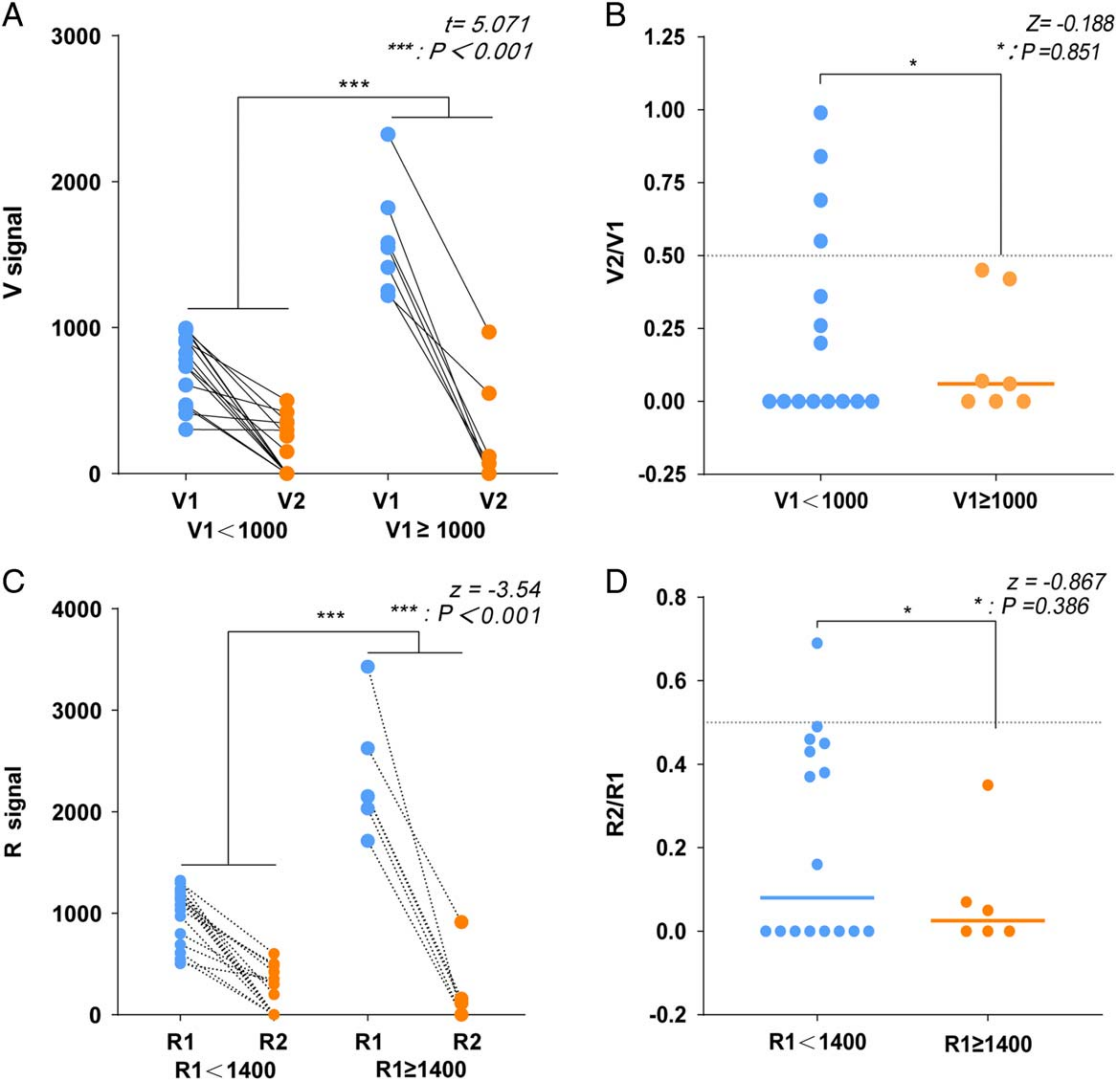
Changes of nerve EMG amplitude in VCP patients. We analyzed and compared intraoperative amplitude changes within higher and lower initial amplitude groups of patients who developed VCP (A–D). The patients were divided into two groups according to high or low initial amplitude with V1=1000 and R1=1400 as a cut-off.

Next, we analyzed and compared intraoperative amplitude changes within higher and lower initial amplitude groups of patients who developed VCP or not. First, the patients with postoperative transient VCP (*n*=22) were divided into two groups according to high or low initial amplitude with V1=1000 as a cut-off. As shown in (Figure 2A, B), 27% (4/15) of the patients with V1<1000 exhibited intraoperative amplitude decreases of less than 50% of the V1 signal (decreases of 1, 16, 31, and 45%, respectively), yet postoperative transient VCP occurred. These results imply that VCP can occur even with small intraoperative amplitude changes when the initial amplitude is low. However, when the V1 signal was greater than 1000, the amplitude decrease was greater than 50% of the V1 signal in all of the VCP patients (*n*=7), with decreases ranging from 55 to 100%. These results are consistent with guideline recommendations and clinical experience. A similar phenomenon was observed in the high or low R1 groups (Fig. 2C, D). Thus, even if the intraoperative amplitude decrease is less than 50%, VCP may still occur when the initial amplitude is low, suggesting that greater attention should be paid to protecting neurological function in patients with low initial amplitude during surgery.

**Figure 3:**
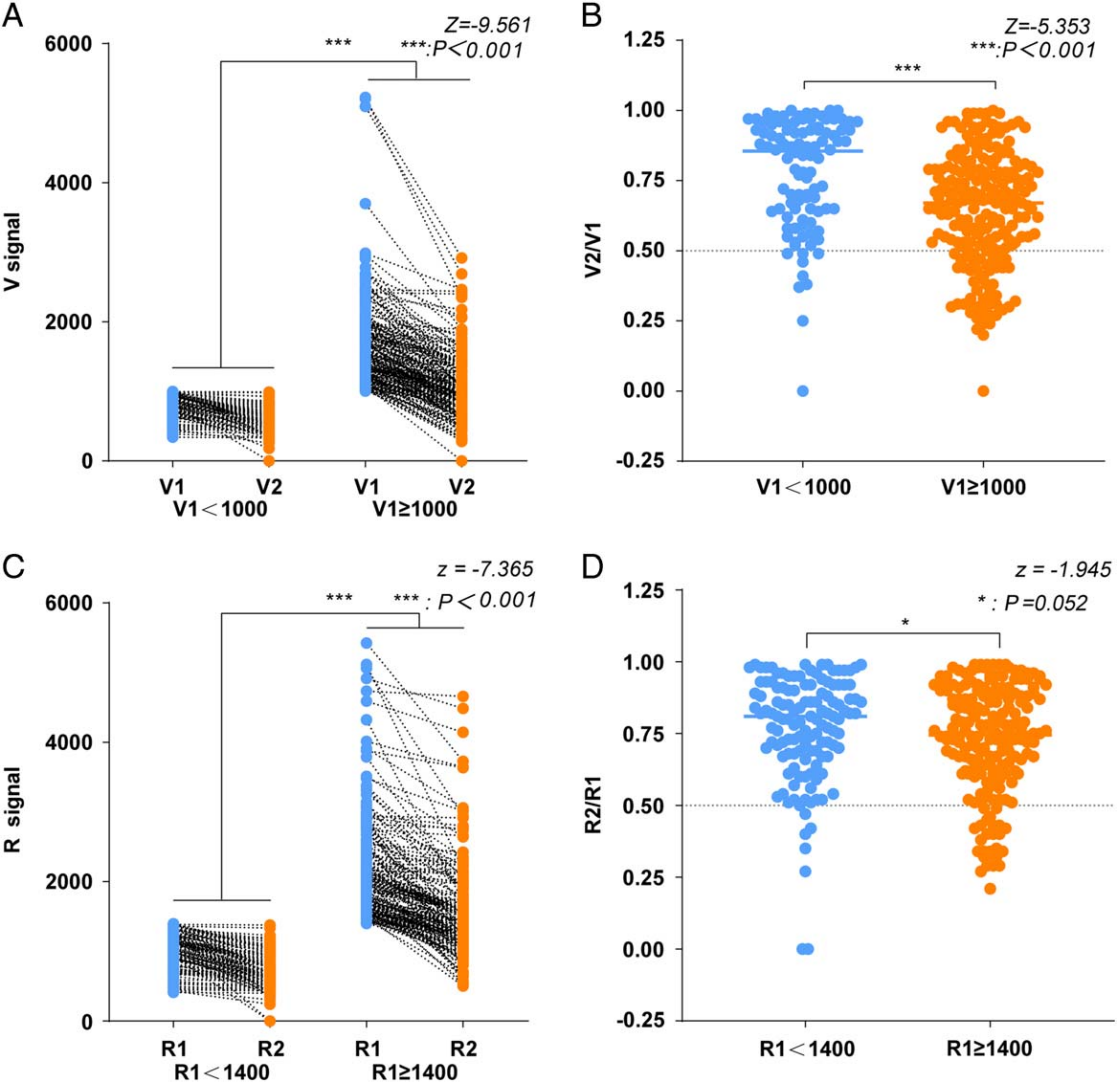
Changes of nerve EMG amplitude in non-VCP patients. We analyzed and compared intraoperative amplitude changes within higher and lower initial amplitude groups of patients who not developed VCP (A–D). The patients were divided into two groups according to high or low initial amplitude with V1=1000 and R1=1400 as a cut-off.

For the patients exhibiting an intraoperative amplitude drop without VCP (Fig. 3A, B), the median intraoperative amplitude decrease [(V1–V2) / V1] for V1<1000 was 15% (*P*_25_–*P*_75_, 6–35%). However, when the V1 signal was greater than 1000, a greater decrease in amplitude was observed, with a median of 33% (*P*_25_–*P*_75_, 21–49%). The magnitude of amplitude change was significantly lower in the V1<1000 group than in the V1 ≥1000 group (*P*<0.001). A greater number of nerves in the V1 ≥1000 group were also able to maintain integrity of nerve function despite their EMG signal being reduced to less than 50% of the V1 signal. Taken together, these results suggest that the amplitude of the nerve in the V1 ≥1000 group was able to undergo a greater decrease, consistent with preservation of nerve function integrity. However, for V1<1000, the amplitude decrease must be minimized to avoid VCP, and not limited to only 50% of the original amplitude. A similar phenomenon was observed in the high or low R1 groups with R1=1400 as the cut-off (Fig. 3C, D). Thus, for patients with low initial amplitude, the surgeon should try to minimize a subsequent decrease in amplitude.

**Figure 4:**
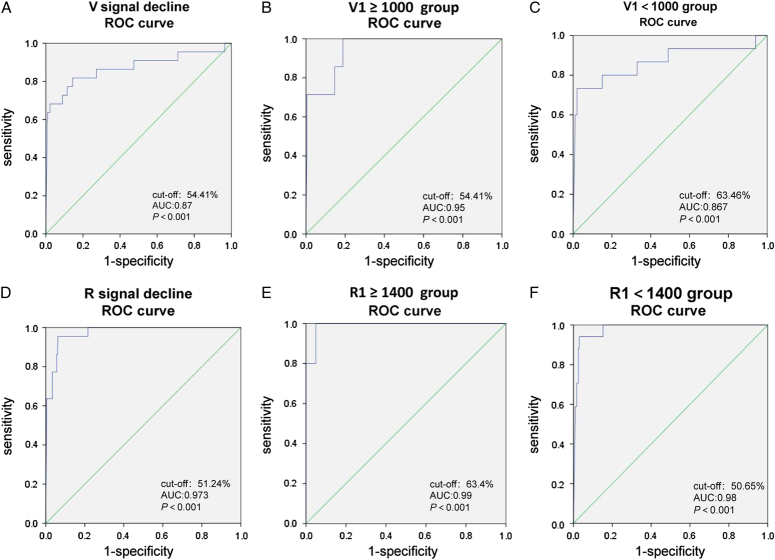
Comparison of the diagnostic ability of VCP with the change of intraoperative amplitude in patients with decreased amplitude. ROC curves were constructed for the two groups with high and low initial amplitudes to assess the predictive value of amplitude change [(V1–V2) / V1] and [(R1–R2) / R1] for postoperative VCP. AUC of the intraoperative V signal decline for predicting postoperative VCP as shown in Figure 4A–C. The AUC value of postoperative VCP diagnosed by intraoperative R signal decline as shown in Figure 4D–F.

ROC curves were constructed for the two groups with high and low initial amplitudes to assess the predictive value of amplitude change [(V1–V2) / V1] and [(R1–R2) / R1] for postoperative VCP. AUC of the intraoperative V signal decline for predicting postoperative VCP was 0.87 (Fig. 4A–C). The predictive accuracy reached as high as 0.95 in the group with V1 ≥1000, yet was greatly reduced (0.867) in the group with V1<1000, indicating that the lower initial amplitude indeed affected the predictive efficacy of intraoperative amplitude changes on postoperative temporary VCP. However, the AUC value of postoperative VCP diagnosed by intraoperative R signal decline reached 0.973 (Fig. 4D–F). In contrast, AUC values of 0.99 and 0.98 were observed for the R1 ≥1400 and R1<1400 groups, respectively.

### Variables influencing low baseline amplitudes

To investigate what factors influence the level of initial amplitude, we first excluded the influence of different anesthetics. Therefore, only patients who were administered cisatracurium were included in our analysis (*n*=454). The corresponding 624 NAR were subsequently grouped according to V1 signal, and possible correlations with clinical characteristics were examined. There were a greater number of male patients in the V1<1000 group compared with the V1 ≥1000 group (32.6 vs. 18.2%, respectively; *P*<0.001). The V1<1000 group also had a higher BMI (25.62 kg/m^2^ vs. 24.39 kg/m^2^, respectively; *P*<0.001), a higher proportion of patients with hypertension (19.2 vs. 9.9%, respectively; *P*=0.001), a higher number of smokers (18.8 vs. 8.5%, respectively; *P*<0.001), and a higher dosage of muscle relaxants used (3.5 mg vs. 3.05 mg, respectively; *P*<0.001) (Table 3). Scatter plots confirmed differences between the subgroups (Supplementary Figure 3, Supplemental Digital Content 2, http://links.lww.com/JS9/C2). In addition, when we enrolled 284 unilateral surgical patients as study participants (Supplementary Table 5, Supplemental Digital Content 2, http://links.lww.com/JS9/C2), except for the hypertension (16.1 vs. 8.8%, respectively; *P*=0.057), the same factors showed relative association as followed: male patients (42.7 vs. 22.5%, respectively; *P*<0.001) and a higher BMI (25.3 kg/m^2^ vs. 24.36 kg/m^2^, respectively; *P*=0.024), as well as a greater proportion of smokers (19.4 vs. 8.8%, respectively; *P*=0.009).

**Table 3:** Analysis of influencing factors of V1 signal-based on nerves at risk.

	Nerves at risk (*n*=624)		Univariate		Multivariate	
	V1<1000 (*n*=261)	V1≥1000 (*n*=363)		*P*	OR (95% CI)	*P*
Age (years), median [IOR]	46 (37–52.5)	45 (36–52)	Z=−0.268	0.789	—	—
Sex, *n* (%)						
Female	176 (67.4)	297 (81.8)	*χ* ^2^=17.129	0.000	1	0.068
Male	85 (32.6)	66 (18.2)			1.516 (0.97–2.37)	
Height (cm), median [IOR]	164 (160–170)	162 (159–167)	Z=−3.854	0.000	—	—
Weight (kg), median [IOR]	69 (60–80)	64 (56–70)	Z=−5.48	0.000	—	—
BMI, mean±SD	25.62 (3.45)	24.39 (3.52)	t=4.341	0.000	1.072 (1.015–1.132)	0.013
BMI, *n* (%)						
<18.5	1 (0.4)	9 (2.5)	Z=−3.41	0.001	—	—
18.5–24.99	121 (46.4)	205 (56.5)				
25–29.99	109 (41.8)	125 (34.4)				
≥30	30 (11.5)	24 (6.6)				
Muscle release dose (mg), median [IOR]	3.5 (3–4)	3.05 (3–4)	Z=−3.333	0.001	0.987 (0.793–1.228)	0.903
Thyroiditis, *n* (%)						
No	240 (92.0)	335 (92.3)	*χ* ^2^=0.023	0.879	—	—
Yes	21 (8.0)	28 (7.7)				
Hyperthyreosis, *n* (%)						
No	250 (95.8)	355 (97.8)	*χ* ^2^=2.079	0.149	—	—
Yes	11 (4.2)	8 (2.2)				
Hypertension, *n* (%)						
No	211 (80.8)	327 (90.1)	*χ* ^2^=10.909	0.001	1	0.015
Yes	50 (19.2)	36 (9.9)			1.816 (1.121–2.942)	
Diabetes, *n* (%)						
No	247 (94.6)	344 (94.8)	*χ* ^2^=0.005	0.943	—	—
Yes	14 (5.4)	19 (5.2)				
Smoking history, *n* (%)						
No	212 (81.2)	332 (91.5)	*χ* ^2^=14.228	0.000	1	0.031
Yes	49 (18.8)	31 (8.5)			1.814 (1.055–3.122)	
Largest tumor size (cm), median [IOR]	0.96 (0.65–1.66)	1.01 (0.72–1.83)	Z=−1.318	0.188	—	

OR, odds ratio.

**Figure 5:**
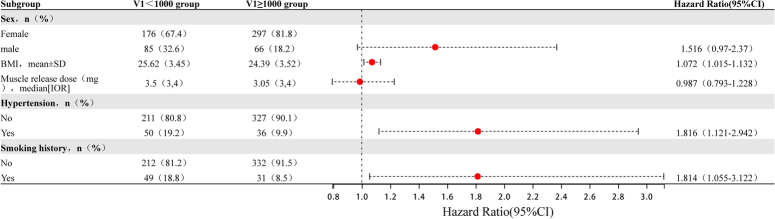
Multivariate Logistic regression forest plots describing the independent risk factors of V1<1000 - based on nerves at risk. To investigate what factors influence the level of initial amplitude, the forest plots of multifactor regression analysis is as shown in Figure 5. The corresponding 624 NAR were grouped according to V1=1000, and possible correlations were examined.

When the above factors were subjected to multivariate logistic regression analysis, the results were shown in Table 3, Figure 5 and Supplementary Table 5, Supplemental Digital Content 2, http://links.lww.com/JS9/C2, Supplementary Figure 2, Supplemental Digital Content 2, http://links.lww.com/JS9/C2, higher BMI (OR=1.072, 95% CI=1.015–1.132, *P*=0.013) combined with hypertension (OR=1.816, 95% CI=1.121–2.942, *P*=0.015), smoking (OR=1.814, 95% CI=1.055–3.122, *P*=0.031), and male sex (OR=2.016, 95% CI=1.085–3.748, *P*=0.027) were identified as independent risk factors for V1<1000. These results suggest that the risk of V1<1000 is higher as BMI increases. In addition, the risk of V1<1000 was two times that of in male patients than in female patients. To further clarify whether different factors influence low V1 signal in either the left or right nerves, a subgroup analysis of patients who underwent unilateral surgery was performed. The factors which influenced the V1 signal on both sides were male gender and higher body weight (Supplementary Table 6, Supplemental Digital Content 2, http://links.lww.com/JS9/C2). Similarly, possible factors influencing low R1 signal were male sex and higher BMI (Supplementary Tables 7–9, Supplemental Digital Content 2, http://links.lww.com/JS9/C2). Taken together, these results suggest that male sex, smoking, combined hypertension, and higher BMI are factors which influence a lower initial amplitude. Therefore, patients with these characteristics should be more closely monitored during thyroid surgery.

## Discussion

Previous studies have investigated the influence of neural signal changes on VCP during surgery^10–13^, as well as the influence of patients’ baseline conditions and postoperative pathological information on VCP^3,22–27^. Based on our clinical experience, we tested the hypothesis that low initial amplitude for V1 and R1 are associated with postoperative VCP.

First, we investigated the correlation between initial amplitude and transient postoperative VCP. In order to screen out the tangent point value suitable for the initial amplitude grouping, V1 and R1 signals were grouped according to the cut-off value obtained from the ROC curve, median method, mean method, and interquartile interval method, respectively. When we grouped R1 according to the median method, this approach resulted in *P-*values being <0.05 and a statistically significant difference was observed, which was better than other statistical methods. In a clinical setting, adjustment of the neural monitor signal threshold to a specific value can be time consuming. It is more convenient to adjust the threshold to a specific integer, therefore, we separately grouped V1 and R1 according to integers near the median. When V1 selected 1000 and R1 selected 1400 for grouping, it is not only statistically significant, but also can be better implemented in clinical practice. Since the RLN originates from the VN, V1, and R1 reflect the same electrophysiological conduction pathway of the nerve. Therefore, given this collinearity, V1=1000 and R1=1400 were each included in a multivariate analysis with other factors, low initial amplitude, and combined thyroiditis were identified as independent risk factors for development of postoperative transient VCP. Regarding the latter, thyroiditis leads to changes in the texture of the thyroid gland, which tend to require additional intraoperative hemostatic procedures during surgery. These additional procedures may lead to RLN damage, consistent with an increased occurrence of VCP. There were a greater number of patients with higher BMI and hypertension in the VCP than in the non-VCP group, consistent with a univariate analysis previously conducted by Gunn *et al.* ^22^ that showed hypertension may be associated with nerve damage. The anatomical features of patients with a higher BMI due to obesity, including a thick and short neck, also increase the difficulty of surgery and are not conducive to protecting nerve function.

According to previous studies and guidelines, an EMG amplitude that drops to 50% of baseline can warn of nerve injury^21,28,29^. Therefore, if the initial amplitude is high, the amplitude can drop further provided that nerve function remains intact. As a result, the nerve exhibits better ‘tolerance’ and is not susceptible to injury. However, if the initial amplitude is low, even if the EMG signal only drops by a few hundred μV, it has already exceeded the safety range and the probability of nerve injury is greatly increased. In the present study, the amplitude among the VCP patients in the higher initial amplitude group decreased to less than 50% of the initial amplitude, which confirmed the reliability of using 50% of the initial amplitude as a warning line to predict nerve injury. Meanwhile, in the lower initial amplitude group, some of the patients that exhibited a low amplitude decrease still experienced transient postoperative VCP. These results suggest that patients with a lower initial amplitude are still susceptible to VCP even when the intraoperative amplitude decrease is less than 50%. And patients with lower initial amplitude have a smaller space for intraoperative amplitude decline and a lower fault tolerance rate for the surgeon. Among the non-VCP patients who experienced a drop in amplitude, a greater number of nerves in the high initial amplitude group had nerve function remain intact even when the EMG amplitude dropped below 50% of the initial amplitude. This result confirms that nerves with higher initial amplitude achieve better ‘tolerance’. Therefore, surgeons should actively communicate with anesthesiologists to follow a standard induction protocol to ensure that the EMG signal as high as possible at the start of surgery. Moreover, when the initial amplitude is low, significant changes in the EMG signal during surgery need to be avoided.

In addition, the ROC curve of intraoperative amplitude decline in predicting the occurrence of postoperative temporary VCP showed that the value of R signal decline was more predictive of postoperative transient VCP than the decline in intraoperative V signal (AUC, 0.973 vs. 0.87, respectively). The diagnostic efficacy of intraoperative R signal decline for postoperative VCP was also better in both R1 high and low groups. The importance of intraoperative real-time monitoring and comparison of RLN amplitude changes was confirmed. Through the discovery of this phenomenon, we believe that if both VN and RLN signals are detected at the beginning of surgery to obtain the R1 ‘signal, and then set the event threshold to 50% of R1’ signal, this may provide early warning of nerve injury and reduce the occurrence of temporary VCP. The R1’ signal can be detected by cutting the linea alba cervicalis, by applying a large spherical probe, and by slowly penetrating the lower pole of the thyroid along the trachea. A 3.0 mA electrical current can be applied for repeated detection in a search for the highest point of the signal. At this point, the RLN signal can be identified according to its waveform and latency, and recorded as the R1’ signal. The event threshold can then be set according to this signal. This method is envisaged based on the present findings. In the future, we will conduct relevant studies to test the effectiveness of this method. Besides, we also found in our subgroup analysis, the predictive power of intraoperative amplitude changes for postoperative VCP was lower in the low V1 group. This suggests that the value of amplitude change for predicting postoperative VCP is not as accurate when the V1 signal amplitude is low. Therefore, more attention should be paid to intraoperative nerve protection when the initial amplitude is low.

Finally, we analyze the influencing factors that might be related to the lower initial amplitude. The V1 signal is the first EMG signal detected during the surgical process, and is currently a classic representation of initial amplitude. Most institutions initially detect the V1 signal according to a standardized protocol. Besides, the diagnostic efficacy of intraoperative R signal decline for postoperative VCP was better in both R1 high and low groups, and the AUC value were higher than 0.95. In contrast, the diagnostic efficacy of V signal decline for VCP in the V1<1000 group was significantly lower than that for a higher V1 signal. Taken together, these results suggest that amplitude change when the V1 signal is low does not perfectly predict VCP. Therefore, we took the V1 signal as the initial amplitude to detect possible factors that could affect its low signal value.

Both univariate and multivariate logistic regression analyses confirmed that a male gender, a history of smoking, hypertension and a higher BMI are independent risk factors for low initial amplitude (V1<1000). Male patients were more represented in the low initial amplitude group and this may be due to a thicker trachea and smaller contact area between the vocal cords and the electrode of the ET tube that characterize male patients versus female patients. Therefore, endotracheal intubation of male patients usually includes use of size 7.0 ET tubes (Medtronic), while size 6.0 ET tubes (Medtronic) are usually used for female patients to ensure the contact between the vocal cords and the electrodes as much as possible. We also observed the effect of BMI on initial amplitude. A higher BMI was associated with a lower initial amplitude, which could be due to the fact that the application of the muscle relaxation dose is related to body weight, patients with a higher BMI tend to have a higher weight. Furthermore, hypertension and smoking were associated with a lower initial amplitude, and this has not been reported in similar studies. And this needs to be further elucidated by analyzing clinical data and pathophysiological experiments. At the same time, we explored the possible influencing factors of the low R1 signal, and the results were similar to those of V signal. The above results suggest that in clinical practise, the surgeon should be vigilant in patients with a male sex, a history of smoking, hypertension, and a higher BMI and try to communicate fully with the anesthetist to avoid low initial amplitudes. In order to achieve a high baseline amplitude, we believe that the most important thing during the operation is the correct placement of the ET tubes and the use of the muscle relaxant according to the standard. In addition, we believe that one of the most important factors affecting the baseline amplitude is the contact between vocal cords and ET tube electrodes. Therefore, in clinical practice, monitoring tubes of appropriate size should be selected according to the diameter of patients’ tracheal tubes to ensure good contact between them and vocal cords. For this reason, cervical ultrasound (including larynx and vocal cord ultrasound) was routinely performed for all surgical patients in our center before surgery, so as to understand the glottis size and vocal cord movement of patients before surgery, and assist in the selection of ET tubes for monitoring. In addition, the anesthesiologist performing the tracheal intubation under a visual laryngoscope is more conducive to maintaining a higher baseline amplitude. During the intubation process, no lubricant or laryngeal spray is used, which also facilitates good contact between the vocal cords and the electrodes and improves the baseline amplitude.

Future studies are also needed to address the limitations associated with the present study. The latter include a small sample size and low incidence of VCP at our center. The results were more convincing when a larger sample size was included for statistical analysis, but the sample size of this study was enough to get a statistically supported conclusion through data analysis. Both of these factors can be addressed with larger sample sizes and by analyzing data from multiple centers in future studies.

## Conclusion

In this study, low initial amplitude was confirmed to be an independent risk factor for postoperative transient VCP in the cohort examined. Moreover, lower baseline amplitude affects the predictive efficacy of intraoperative amplitude changes on VCP, and patients with low initial amplitude should be closely monitored to protect their neurological function.

It was further observed that male patients, patients with a history of smoking, and patients with hypertension or obesity are more likely to exhibit low baseline amplitudes. Thus, greater attention to anesthesia induction and intraoperative protection of nerve function in these patients by anesthesiologists and surgeons is needed to achieve good baseline amplitude if possible. Finally, the importance of intraoperative real-time monitoring and comparison of RLN EMG changes were confirmed.

## Ethical approval

The study protocol was approved by the Ethics Committee of China-Japan Union Hospital, Jilin University (Protocol reference number: 2023020719).

## Consent

Written informed consent was obtained from the patient for publication of this case report and accompanying images. A copy of the written consent is available for review by the Editor-in-Chief of this journal on request.

## Sources of funding

This study was sponsored by the Jilin Province Science and Technology Development Project [YDZJ202201ZYTS112]; the project of China-Japan Union Hospital [2023CL01]; the Project of Jilin Provincial Finance Department [2023SCZ26;2023SCZ51];Science and Technology Research Project of Education Department of Jilin Province, China,[No.JJKH20221065KJ]; Jilin Province Health Research Talent Special Project[No.2020SCZ03]; Beijing Cihua Medical Development Foundation[J2023107004].We would also like to thank the many clinicians in the department for their support in data collection.

## Author contribution

H.S. and Y.S.Z.: conceptualization; J.D.K., Y.J.H., F.L., and R.D.: methodology; J.D.K. and Y.S.Z: formal analysis; J.D.K. and Y.J.H: investigation; J.D.K., Y.S.Z., Y.J.H., and F.L.: resources; J.D.K. and Y.S.Z.: writing – original draft preparation; N.L., H.S., G.D., and F.F.: writing – review and editing; H.S.: supervision; N.L., Y.S.Z., and H.S.: project administration; N.L.,Y.S.Z., and H.S.: funding acquisition. All authors have read and agreed to the published version of the manuscript.

## Conflicts of interest disclosure

The authors declared no conflicts of interest.

## Research registration unique identifying number (UIN)

The unique identifying number is: researchregistry9789, and you will find your registration here: https://www.researchregistry.com/browse-the-registry#home/.

## Guarantor

All authors have read and agreed to the published version of the manuscript.

## Data availability statement

Please refer to the corresponding author when necessary.

## Provenance and peer review

This paper was not invited, externally peer-reviewed.

## Supplementary Material
