# Supplementary material for: Noticeable effect of lower baseline amplitude on the predictive accuracy of intraoperative amplitude changes for postoperative vocal cord palsy: a prospective cohort study
Source: Int J Surg. 2024 Feb 21;110(5):2765–75. doi: 10.1097/JS9.0000000000001203 (PMC11093453; doi:10.1097/JS9.0000000000001203)
Supplement: Supplementary file 2 [file js9-110-2765-s002.docx]

**Noticeable effect of lower baseline amplitude on the predictive accuracy of intraoperative amplitude changes for postoperative vocal cord palsy: A prospective cohort study**

Jiedong Kou^1,#^, Yishen Zhao^1,#^, Yujia Han^1^, Fang Li^1^, Rui Du^1^, Gianlorenzo Dionigi ^2^, Francesco Frattini ^2^, Jingting Li^1^, Hui Sun ^1,*^, Nan Liang^1, *^

| **Supplementary** **table 1.** **Standardization of IONM in thyroid surgery** | |
| --- | --- |
| L1 | Pre-operative laryngoscopy |
| V1 | Test vagus nerve before identification of RLN |
| R1 | Test RLN when it was identified at the tracheo-esophageal grove |
| R2 | Test RLN after it was completely dissected from Berry's ligament |
| V2 | Test vagus nerve after complete hemostasis |
| L2 | Post-operative laryngoscopy |
| IONM = intraoperative neural monitoring; RLN = recurrent laryngeal nerve. | |
| \| **Supplementary** **table 2. Verification test for proper contact of the EMG tube with the vocal cords** \| \| \| --- \| --- \| \| Preoperatively \| 1) The impedance values readable on the IONM monitor, it should be less than 5 kΩ per electrode \| \| 2) Repeat laryngeal examination after patient head extension to check possible tube desplacemnet \| \| 3) Respiratory variation \| \| 4) Tap test \| \| Intraoperatively \| 5) Achieve intraoperative V1 as high as possible \| \| 6) All above \| \| EMG = electromyographic; IONM = intraoperative neuromonitoring.   \| **Supplementary** **table 3. Definition of loss of EMG signal** \| \| --- \| \| • Normal vocal cord movement at preoperative laryngeal examination (L1) \| \| • Initial satisfactory EMG signal (V1 >500 mV) \| \| • No EMG response with stimulation at 1–2 mA \| \| • Low response <100 mV with stimulation at 1–2 mA \| \| • No LT \| \| • Troubleshooting algorithm applied systematically \| \| EMG = electromyographic; LT = laryngeal twitch. \| \| \| | |

**Supplementary Table 4. Correlation analysis between initial amplitude and vocal cord palsy**

|  | **Non-VCP**  **（n=609）** | **VCP（n=22）** |  | ***P*-value** | |  | **Non-VCP**  **（n=609）** | **VCP（n=22）** |  | ***P-*value** |
| --- | --- | --- | --- | --- | --- | --- | --- | --- | --- | --- |
| **V1（quantitative），median[IOR]** | 1120（796,1600） | 950.5（700.8,1293） | Z= -1.873 | 0.061 | **R1（quantitative），median[IOR]** | | 1448（1020,2009） | 1151.5（927,1500.5） | Z= -1.779 | 0.075 |
| **V1（cut-off），n（%）** |  |  |  |  | **R1（cut-off），n（%）** | |  |  |  |  |
| ≤299.5 | 3（0.5） | 0（0.5） | Fisher | 1.000 | ＜502 | | 18（3.0） | 0（0） | Fisher | 1.000 |
| ＞299.5 | 606（99.5） | 22（100.0） |  |  | ≥502 | | 591（97.0） | 22（100） |  |  |
| **V1（statistic），n（%）** |  |  |  |  | **R1（statistic），n（%）** | |  |  |  |  |
| ﹤1000 | 248（40.7） | 15（68.2） | χ2= 6.586 | 0.014 | ＜1400 | | 286（47.0） | 17（77.3） | χ2= 7.815 | 0.005 |
| ≥1000 | 361（59.3） | 7（31.8） |  |  | ≥1400 | | 323（53.0） | 5（22.7） |  |  |
| **V1（median），n（%）** |  |  |  |  | **R1（median），n（%）** | |  |  |  |  |
| ≤1114 | 300（49.3） | 15（68.2） | χ2=3.041 | 0.081 | ＜1429 | | 297（48.8） | 17（77.3） | χ2= 6.901 | 0.009 |
| ＞1114 | 309（50.7） | 7（31.8） |  |  | ≥1429 | | 312（51.2） | 5（22.7） |  |  |
| **V1（mean value），n（%）** |  |  |  |  | **R1（mean value），n（%）** | |  |  |  |  |
| ≤1261.2 | 361（59.3） | 17（77.3） | χ2= 2.863 | 0.091 | ＜1584 | | 359（58.9） | 17（77.3） | χ2= 2.96 | 0.085 |
| ＞1261.2 | 248（40.7） | 5（22.7） |  |  | ≥1584 | | 250（41.1） | 5（22.7） |  |  |
| **V1（interquartile），n（%）** |  |  |  |  | **R1（interquartile），n（%）** | |  |  |  |  |
| ＜789 | 149（24.5） | 8（36.4） | Z= -1.757 | 0.079 | ＜1020 | | 151（24.8） | 6（27.3） | Z= -1.376 | 0.169 |
| 789～1113.99 | 151（24.8） | 7（31.8） |  |  | 1020～1428.99 | | 146（24.0） | 11（50） |  |  |
| 1114～1574.99 | 154（25.3） | 4（18.2） |  |  | 1429～2007.99 | | 159（26.1） | 0（0） |  |  |
| ≥1575 | 155（25.5） | 3（13.6） |  |  | ≥2008 | | 153（25.1） | 5（22.7） |  |  |

**Supplementary Table 5. Analysis of influencing factors of V1 signal - based on unilateral surgical patients**

|  | **Unilateral surgical patients（n=284）** | | **Univariate** | | **Multivariate** | |
| --- | --- | --- | --- | --- | --- | --- |
|  | **V1＜1000（n=124）** | **V1≥1000（n=160）** |  | ***P*-value** | **OR（95%CI）** | ***P*-value** |
| **Age（year），mean±SD** | 42.48（10.27） | 43.21（10.01） | t= 0.601 | 0.548 | — | — |
| **Sex，n（%）** |  |  |  |  |  |  |
| Female | 71（53.7） | 124（77.5） | χ2=13.303 | 0.000 | 1 | 0.027 |
| male | 53（42.7） | 36（22.5） |  |  | 2.016（1.085-3.748） |  |
| **Height（cm），median[IOR]** | 166（160,171.5） | 163（160,168） | Z= -2.855 | 0.004 | — | — |
| **Weight（kg），median[IOR]** | 69（60,80） | 64.5（57,72.38） | Z= -3.215 | 0.001 | — | — |
| **BMI，mean±SD** | 25.30（3.40） | 24.36（3.51） | t= 2.275 | 0.024 | 1.035（0.959-1.117） | 0.375 |
| **BMI，n（%）** |  |  |  |  |  |  |
| ﹤18.5 | 1（0.8） | 5（3.1） | Fisher | 0.368 | — | — |
| 18.5-24.99 | 59（47.6） | 85（53.1） |  |  |  |  |
| 25-29.99 | 55（44.4） | 61（38.1） |  |  |  |  |
| ≥30 | 9（7.3） | 9（5.6） |  |  |  |  |
| **Muscle release dose（mg），median[IOR]** | 3.38（3,4） | 3（3,3.9） | Z= -1.674 | 0.094 | — | — |
| **Thyroiditis，n（%）** |  |  |  |  |  |  |
| No | 110（88.7） | 147（91.9） | χ2=0.814 | 0.367 | — | — |
| Yes | 14（11.3） | 13（8.1） |  |  |  |  |
| **Hyperthyreosis，n（%）** |  |  |  |  |  |  |
| No | 123（99.2） | 160（100） | Fisher | 0.437 | — | — |
| Yes | 1（0.8） | 0（0） |  |  |  |  |
| **Hypertension，n（%）** |  |  |  |  |  |  |
| No | 104（83.9） | 146（91.3） | χ2=3.609 | 0.057 | — | — |
| Yes | 20（16.1） | 14（8.8） |  |  |  |  |
| **Diabetes，n（%）** |  |  |  |  |  |  |
| No | 115（92.7） | 154（96.3） | χ2=1.719 | 0.19 | — | — |
| Yes | 9（7.3） | 6（3.8） |  |  |  |  |
| **Smoking history，n（%）** |  |  |  |  |  |  |
| No | 100（80.6） | 146（91.3） | χ2=6.779 | 0.009 | 1 | 0.302 |
| Yes | 24（19.4） | 14（8.8） |  |  | 1.522（0.686-3.38） |  |
| **Largest tumor size（cm），median[IOR]** | 0.84（0.62,1.24） | 0.85（0.63，1.42） | Z= -0.771 | 0.441 | — | — |

**Supplementary table 6.** **Analysis of influencing factors of low V1 signal in patients with unilateral operation**

|  | **Left side operation（n=138）** | | | | **Right side operation（n=146）** | | | |
| --- | --- | --- | --- | --- | --- | --- | --- | --- |
|  | **V1＜1000（n=67）** | **V1≥1000（n=71）** |  | ***P*-value** | **V1＜1000（n=57）** | **V1≥1000（n=89）** |  | ***P*-value** |
| **Age（year），mean±SD/median[IOR]** | 42（34，52） | 43（33,51） | Z= -0.518 | 0.604 | 41.35（9.95） | 43.82（9.98） | t= -0.146 | 0.146 |
| **Sex，n（%）** |  |  |  |  |  |  |  |  |
| Female | 36（53.7） | 51（71.8） | χ2=4.847 | 0.028 | 35（61.4） | 73（82.0） | χ2=7.673 | 0.006 |
| male | 31（46.3） | 20（28.2） |  |  | 22（38.6） | 16（18.0） |  |  |
| **Height（cm），median[IOR]** | 167（160,172） | 164（160,170） | Z= -1.458 | 0.145 | 166（160,170） | 163（160,166） | Z= -2.469 | 0.014 |
| **Weight（kg），median[IOR]** | 69（60,80） | 62（55,75） | Z= -2.134 | 0.033 | 70（60,81） | 65（59,70） | Z= -2.415 | 0.016 |
| **BMI，mean±SD/median[IOR]** | 25.38（22.66，27.34） | 24.03（21.23，26.83） | Z= -1.791 | 0.073 | 25.44（3.49） | 24.45（3.31） | t=1.717 | 0.088 |
| **Muscle release dose（mg），median[IOR]** | 3.25（3,4） | 3（3,4） | Z= -1.587 | 0.113 | 3.5（3,4） | 3（3,3.63） | Z= -0.766 | 0.444 |
| **Thyroiditis，n（%）** |  |  |  |  |  |  |  |  |
| No | 57（85.1） | 64（90.1） | χ2=0.819 | 0.365 | 53（93.0） | 83（93.3） | Fisher | 1.00 |
| Yes | 10（14.9） | 7（9.9） |  |  | 4（7.0） | 6（6.7） |  |  |
| **Hyperthyreosis，n（%）** |  |  |  |  |  |  |  |  |
| No | 67（100） | 71（100） | / | / | 56（98.2） | 89（100） | Fisher | 0.39 |
| Yes | 0（0） | 0（0） |  |  | 1（1.8） | 0（0） |  |  |
| **Hypertension，n（%）** |  |  |  |  |  |  |  |  |
| No | 55（82.1） | 65（91.5） | χ2=2.72 | 0.099 | 49（86.0） | 81（91.0） | χ2=0.907 | 0.341 |
| Yes | 12（17.9） | 6（8.5） |  |  | 8（14.0） | 8（9.0） |  |  |
| **Diabetes，n（%）** |  |  |  |  |  |  |  |  |
| No | 60（89.6） | 69（97.2） | Fisher | 0.09 | 55（96.5） | 85（95.5） | Fisher | 1.00 |
| Yes | 7（10.4） | 2（2.8） |  |  | 2（3.5） | 4（4.5） |  |  |
| **Smoking history，n（%）** |  |  |  |  |  |  |  |  |
| No | 55（82.1） | 66（93.0） | χ2=3.77 | 0.052 | 45（78.9） | 80（89.9） | χ2=3.377 | 0.066 |
| Yes | 12（17.9） | 5（7.0） |  |  | 12（21.1） | 9（10.1） |  |  |
| **Largest tumor size（cm），median[IOR]** | 0.81（0.63,1.23） | 0.9（0.65，1.5） | Z= -0.788 | 0.431 | 0.84（0.60,1.28） | 0.82（0.62，1.37） | Z= -0.327 | 0.744 |

|  | **Nerves at risk（n=624）** | | **Univariate** | | **Multivariate** | |
| --- | --- | --- | --- | --- | --- | --- |
|  | **R1＜1400（n=301）** | **R1≥1400（n=323）** |  | ***P*-value** | **OR（95%CI）** | ***P*-value** |
| **Age（year），median[IOR]** | 46（38,53） | 45（35,52） | Z= -1.361 | 0.173 | — | — |
| **Sex，n（%）** |  |  |  |  |  |  |
| Female | 210（69.8） | 263（81.4） | χ2=11.542 | 0.001 | 1 | 0.046 |
| male | 91（30.2） | 60（18.6） |  |  | 1.521（1.007-2.296） |  |
| **Height（cm），median[IOR]** | 164（160,170） | 162（158,167） | Z= -2.917 | 0.004 | — | — |
| **Weight（kg），median[IOR]** | 68（60,79.5） | 64（56,70） | Z= -5.151 | 0.000 | — | — |
| **BMI， median[IOR]** | 25.24（23.36，27.78） | 24.01（21.77，26.37） | Z= -4.626 | 0.000 | 1.078（1.021-1.137） | 0.006 |
| **BMI，n（%）** |  |  |  |  |  |  |
| ﹤18.5 | 2（0.7） | 8（2.5） | Z= -2.752 | 0.006 | — | — |
| 18.5-24.99 | 145（48.2） | 181（56） |  |  |  |  |
| 25-29.99 | 122（40.5） | 112（34.7） |  |  |  |  |
| ≥30 | 32（10.6） | 22（6.8） |  |  |  |  |
| **Muscle release dose（mg），median[IOR]** | 3.5（3,4） | 3.1（3,4） | Z= -2.792 | 0.005 | 1.052（0.848-1.305） | 0.644 |
| **Thyroiditis，n（%）** |  |  |  |  |  |  |
| No | 279（92.7） | 296（91.6） | χ2=0.0237 | 0.626 | — | — |
| Yes | 22（7.3） | 27（8.4） |  |  |  |  |
| **Hyperthyreosis，n（%）** |  |  |  |  |  |  |
| No | 289（96.0） | 316（97.8） | χ2=1.747 | 0.186 | — | — |
| Yes | 12（4.0） | 7（2.2） |  |  |  |  |
| **Hypertension，n（%）** |  |  |  |  |  |  |
| No | 249（82.7） | 289（89.5） | χ2=5.973 | 0.015 | 1 | 0.139 |
| Yes | 52（17.3） | 34（10.5） |  |  | 1.44（0.888-2.333） |  |
| **Diabetes，n（%）** |  |  |  |  |  |  |
| No | 286（95） | 305（94.4） | χ2=0.108 | 0.742 | — | — |
| Yes | 15（5） | 18（5.6） |  |  |  |  |
| **Smoking history，n（%）** |  |  |  |  |  |  |
| No | 256（85） | 288（89.2） | χ2=2.36 | 0.125 | — | — |
| Yes | 45（15） | 35（10.8） |  |  |  |  |
| **Largest tumor size（cm），median[IOR]** | 0.96（0.66,1.69） | 1.02（0.69，1.92） | Z= -0.7 | 0.484 | — | — |

**Supplementary table 7.** **Analysis of influencing factors of R1 signal - based on nerves at risk**

|  | **Unilateral surgical patients（n=284）** | | | **Univariate** | | | **Multivariate** | |
| --- | --- | --- | --- | --- | --- | --- | --- | --- |
|  | **R1＜1400（n=141）** | **R1≥1400（n=143）** | |  | ***P*-value** | | **OR（95%CI）** | ***P*-value** |
| **Age（year），mean±SD** | 43.93（10.42） | | 41.87（9.74） | t= -1.717 | | 0.087 | — | — |
| **Sex，n（%）** |  | |  |  | |  |  |  |
| Female | 83（58.9） | | 112（78.3） | χ2=12.49 | | 0.000 | 1 | 0.023 |
| male | 58（41.1） | | 31（21.7） |  |  |  | 1.978（1.099-3.559） |  |
| **Height（cm），median[IOR]** | 165（160,170） | | 163（160,168） | Z= -2.031 | | 0.042 | — | — |
| **Weight（kg），median[IOR]** | 68（60,80） | | 63（57,73） | Z= -3.531 | | 0.000 | — | — |
| **BMI，mean±SD** | 25.36（3.39） | | 24.19（3.49） | t= -2.866 | | 0.004 | 1.501（0.997-1.139） | 0.225 |
| **BMI，n（%）** |  | |  |  | |  |  |  |
| ﹤18.5 | 1（0.7） | | 5（3.5） | Fisher | | 0.354 | — | — |
| 18.5-24.99 | 69（48.9） | | 75（52.4） |  |  |  |  |  |
| 25-29.99 | 61（43.3） | | 55（38.5） |  |  |  |  |  |
| ≥30 | 10（7.1） | | 8（5.6） |  |  |  |  |  |
| **Muscle release dose（mg），median[IOR]** | 3.5（3,4） | | 3（3,3.75） | Z= -2.211 | | 0.027 | 1.139（0.824,1.575） | 0.431 |
| **Thyroiditis，n（%）** |  | |  |  | |  |  |  |
| No | 128（90.8） | | 129（90.2） | χ2=0.027 | | 0.87 | — | — |
| Yes | 13（9.2） | | 14（9.8） |  |  |  |  |  |
| **Hyperthyreosis，n（%）** |  | |  |  | |  |  |  |
| No | 140（99.3） | | 143（100） | Fisher | | 0.496 | — | — |
| Yes | 1（0.7） | | 0（0） |  |  |  |  |  |
| **Hypertension，n（%）** |  | |  |  | |  |  |  |
| No | 120（85.1） | | 130（90.9） | χ2=2.268 | | 0.132 | — | — |
| Yes | 21（14.9） | | 13（9.1） |  |  |  |  |  |
| **Diabetes，n（%）** |  | |  |  | |  |  |  |
| No | 131（92.9） | | 138（96.5） | χ2=1.835 | | 0.176 | — | — |
| Yes | 10（7.1） | | 5（3.5） |  |  |  |  |  |
| **Smoking history，n（%）** |  | |  |  | |  |  |  |
| No | 117（83） | | 129（90.2） | χ2=3.203 | | 0.074 | — | — |
| Yes | 24（17） | | 14（9.8） |  |  |  |  |  |
| **Largest tumor size（cm），median[IOR]** | 0.84（0.64,1.2） | | 0.84（0.6,1.5） | Z= -0.335 | | 0.737 | — | — |

**Supplementary table 8.** **Analysis of influencing factors of R1 signal - based on unilateral surgical patients**

**Supplementary table 9.** **Analysis of influencing factors of low R1 signal in patients with unilateral operation**

|  | **Left side operation（n=138）** | | | | | | | **Right side operation（n=146）** | | | |
| --- | --- | --- | --- | --- | --- | --- | --- | --- | --- | --- | --- |
|  | **R1＜1400（n=70）** | | **R1≥1400（n=68）** | |  | | ***P*-value** | **R1＜1400（n=71）** | **R1≥1400（n=75）** |  | ***P*-value** |
| **Age（year），mean±SD/median[IOR]** | 44（34，53） | 42（33,48） | | Z= -1.739 | | 0.082 | | 43.22（9.60） | 42.51（10.44） | t= -0.432 | 0.666 |
| **Sex，n（%）** |  |  | |  | |  | |  |  |  |  |
| Female | 36（51.4） | 51（75） | | χ2=8.226 | | 0.004 | | 47（66.2） | 61（81.3） | χ2=4.34 | 0.037 |
| male | 34（48.6） | 17（25） | |  |  |  |  | 24（33.8） | 14（18.7） |  |  |
| **Height（cm），median[IOR]** | 167.5（160,172） | 163.5（160,170） | | Z= -1.584 | | 0.113 | | 164（160,170） | 163（160,167） | Z= -1.08 | 0.28 |
| **Weight（kg），median[IOR]** | 69.5（60,80） | 61（56,74.5） | | Z= -2.643 | | 0.008 | | 66（60,78） | 64（57,70） | Z= -2.232 | 0.026 |
| **BMI，mean±SD** | 25.37（3.35） | 24（3.69） | | t= -2.284 | | 0.024 | | 25.35（3.46） | 24.36（3.31） | t=-1.766 | 0.08 |
| **Muscle release dose（mg），median[IOR]** | 3.5（3,4） | 3（3,3.98） | | Z= -1.507 | | 0.132 | | 3.5（3,4） | 3（3,3.5） | Z= -1.578 | 0.115 |
| **Thyroiditis，n（%）** |  |  | |  | |  | |  |  |  |  |
| No | 60（85.7） | 61（89.7） | | χ2=0.509 | | 0.476 | | 68（95.8） | 68（90.7） | Fisher | 0.328 |
| Yes | 10（14.3） | 7（10.3） | |  |  |  |  | 3（4.2） | 7（9.3） |  |  |
| **Hyperthyreosis，n（%）** |  |  | |  | |  | |  |  |  |  |
| No | 70（100） | 68（100） | | / | | / | | 70（98.6） | 75（100） | Fisher | 0.486 |
| Yes | 0（0） | 0（0） | |  |  |  |  | 1（1.4） | 0（0） |  |  |
| **Hypertension，n（%）** |  |  | |  | |  | |  |  |  |  |
| No | 57（81.4） | 63（92.6） | | χ2=3.827 | | 0.075/0.05 | | 63（88.7） | 67（89.3） | χ2=0.013 | 0.908 |
| Yes | 13（18.6） | 5（7.4） | |  |  |  |  | 8（11.3） | 8（10.7） |  |  |
| **Diabetes，n（%）** |  |  | |  | |  | |  |  |  |  |
| No | 63（90） | 66（97.1） | | Fisher | | 0.166 | | 68（95.8） | 72（96） | Fisher | 1.00 |
| Yes | 7（10） | 2（2.9） | |  |  |  |  | 3（4.2） | 3（4） |  |  |
| **Smoking history，n（%）** |  |  | |  | |  | |  |  |  |  |
| No | 60（85.7） | 61（89.7） | | χ2=0.509 | | 0.476 | | 57（80.3） | 68（90.7） | χ2=3.194 | 0.074 |
| Yes | 10（14.3） | 7（10.3） | |  |  |  |  | 14（19.7） | 7（9.3） |  |  |
| **Largest tumor size（cm），median[IOR]** | 0.85（0.64,1.16） | 0.92（0.62，1.63） | | Z= -0.831 | | 0.406 | | 0.83（0.63,1.31） | 0.84（0.58,1.36） | Z= -0.292 | 0.771 |

**Supplementary Figure 1. Distribution of initial amplitude of nerves in VCP and non-VCP groups**


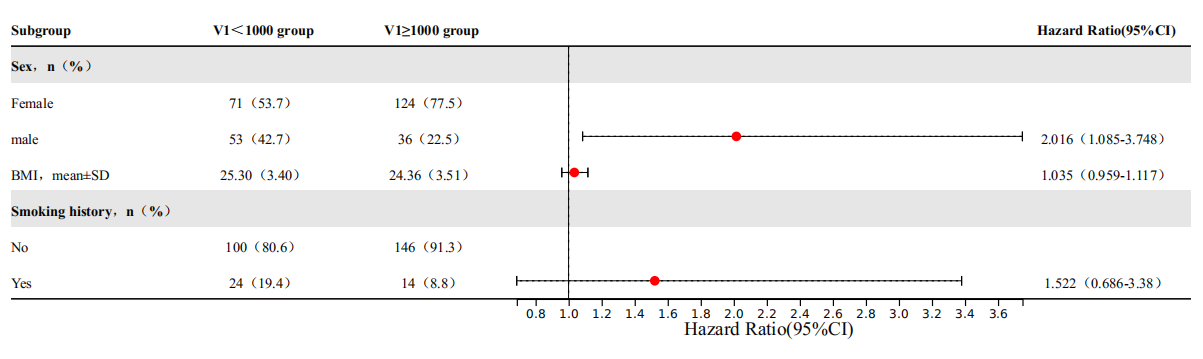


**Supplement Figure 2. Multivariate Logistic regression forest plots describing the independent risk factors of V1 < 1000 - based on unilateral surgical patients**

**Supplementary Figure 3. Distribution of initial amplitudes between different subgroups**
